# Supplementary material for: Content-rich biological network constructed by mining PubMed abstracts
Source: BMC Bioinformatics. 2004 Oct 8;5:147. doi: 10.1186/1471-2105-5-147 (PMC528731; doi:10.1186/1471-2105-5-147)
Supplement: Additional File 2 — The original results of the above study (non-essential files are deleted to keep the file size under the limit set by BMC bioinformatics). [file 1471-2105-5-147-S2.bz2 › chilibotAdditionalFile2/dip30/336ID10768840E3972/html/TNFSF8_TNFRSF8.html]

 


 **TNFSF8** and **TNFRSF8** 
  
Found 158 abstracts in PubMed, retrieved 30.  
 

 What does Google say? 
 PDF only 
| .edu only 

---

**Interactive relationship** (e.g. stimulation, inhibition, etc)

**Neutral relationship**- they activate HRS in vitro through CD30L  [ **TNFSF8** ]  CD30  [ **TNFRSF8** ]  interaction.  Ref: 12358914 Br J Haematol, 2002
- CD30L  [ **TNFSF8** ]  up regulates CD30  [ **TNFRSF8** ]  and IL 4 expression by T cells.  Ref: 11728464 FEBS Lett, 2001
- Human angiogenin fused to human CD30  [ **TNFRSF8** ]  ligand  [ **TNFSF8** ]  Ang CD30L  [ **TNFSF8** ]  exhibits specific cytotoxicity against CD30  [ **TNFRSF8** ]  positive lymphoma.  Ref: 11751393 Cancer Res, 2001
- These interactions include those of CD30L  [ **TNFSF8** ]  CD30  [ **TNFRSF8** ] , CD40L CD40, CD70 CD27, CD80 CD86 CD28, SCF CD117, IL 9 IL 9R, and IL 7 IL 7R.  Ref: 11725067 J Biomed Sci< MedlineTA>J Biomed Sci, 2001
- Characterization of the immunological properties of vCD30 has uncovered a role of CD30  [ **TNFRSF8** ]  CD30L  [ **TNFSF8** ]  interactions in the generation of inflammatory responses.  Ref: 12235215 J Exp Med, 2002
- Soluble trimeric CD153  [ **TNFSF8** ]  bound to membrane anchored CD30  [ **TNFRSF8** ]  with a relatively high affinity K D =23 nM and was effective in triggering cell death and TNF alpha production in the presence of cross linking antibodies.  Ref: 11754357 Eur J Immunol, 2002
- We have generated soluble recombinant forms of CD30  [ **TNFRSF8** ]  and CD153  [ **TNFSF8** ]  in order to characterize their interaction and assess their biological activity.  Ref: 11754357 Eur J Immunol, 2002
- The pleiotropic functions of CD30  [ **TNFRSF8** ]  are initiated by interaction of CD30  [ **TNFRSF8** ]  expressing cells with other immune competent cells expressing CD30  [ **TNFRSF8** ]  L  [ **TNFSF8** ]  and providing the signals for modulation of effector cell activity.  Ref: 12473266 Cell Immunol, 2002
- Specific binding activity of Ang CD30L  [ **TNFSF8** ]  was verified by competition with anti CD30  [ **TNFRSF8** ]  monoclonal antibody Ki 4 and commercially available CD30L  [ **TNFSF8** ]  CD8 chimeric protein.  Ref: 11751393 Cancer Res, 2001
- Comparison of effects of ligation with CD30L  [ **TNFSF8** ]  and anti CD30  [ **TNFRSF8** ]  agonistic antibody HeFi 1 revealed higher efficacy for CD30L  [ **TNFSF8** ]  in these ALCL lines.  Ref: 12855655 Clin Cancer Res, 2003
- However, CD30  [ **TNFRSF8** ]  is cleaved at the cell surface by tumor necrosis factor  [ **TNFSF8** ]  alpha converting enzyme TACE.  Ref: 11857410 Int J Cancer, 2002
- Direct sequencing analysis of the CD30  [ **TNFRSF8** ]  and CD30L  [ **TNFSF8** ]  genes revealed four polymorphisms one in the CD30  [ **TNFRSF8** ]  gene 201G A from the transcription start site, and three in the CD30L  [ **TNFSF8** ]  gene CA repeat in the promoter, 276G A in the exon 3T C in the intron 3 IVS3 73T C.  Ref: 11960307 Genes Immun, 2002
- Furthermore, a number of Th2 associated markers, including CD30  [ **TNFRSF8** ] , IL 4 and GATA 3, were expressed by residual T cells derived from CD30L  [ **TNFSF8** ]  AML but not from CD30L  [ **TNFSF8** ]  AML, in which the presence of the Th1 associated marker LAG 3 was documented in some cases.  Ref: 11918534 Br J Haematol, 2002
- Co expression of CD30  [ **TNFRSF8** ]  ligand  [ **TNFSF8** ]  and interleukin 4 IL 4 receptors by acute myeloid leukaemia blasts is associated with the expansion of IL 4 producing CD30  [ **TNFRSF8** ]  normal T cells.  Ref: 11918534 Br J Haematol, 2002
- We report that in H RS cells overexpression of CD30  [ **TNFRSF8** ]  leads to self aggregation, recruitment of TRAF2 and TRAF5, and NF kappaB activation, independent of CD30  [ **TNFRSF8** ]  ligand  [ **TNFSF8** ] .  Ref: 11971184 Oncogene, 2002
- The tumor necrosis factor  [ **TNFSF8** ]  receptor associated factor 1 TRAF1 participates in the signal transduction of various members of the tumor necrosis factor  [ **TNFSF8** ]  receptor TNFR family, including TNFR2, CD40, CD30  [ **TNFRSF8** ] , and the Epstein Barr virus EBV encoded latent membrane protein 1 LMP1.  Ref: 11567226 Hum Pathol, 2001
- The activation antigen CD30  [ **TNFRSF8** ]  Ki 1  [ **TNFRSF8** ]  which is expressed by the lymphocytes T and B is related to the nerve growth factor NFGR like the receptor of the tumor necrosis factor  [ **TNFSF8** ]  TNF and is expressed in the group of large cell anaplastic lymphoma ACLC and Hodgkin s lymphoma and in non Hodgkin s lymphoma and within the T cells of infectious Mononucleosis.  Ref: 12730648 Infez Med< MedlineTA>Infez Med, 2001
- Soluble CD30  [ **TNFRSF8** ]  binds to CD153  [ **TNFSF8** ]  with high affinity and blocks transmembrane signaling by CD30  [ **TNFRSF8** ] .  Ref: 11754357 Eur J Immunol, 2002
- Proliferation of CD30  [ **TNFRSF8** ]  T Helper 2 Lymphoma Cells Can Be Inhibited by CD30  [ **TNFRSF8** ]  Receptor Cross Linking with Recombinant CD30  [ **TNFRSF8** ]  Ligand  [ **TNFSF8** ] .  Ref: 12855655 Clin Cancer Res, 2003
- In contrast to other members of the TNF receptor superfamily, soluble monomeric CD30  [ **TNFRSF8** ]  bound to its ligand with high affinity K D =4.5 nM and prevented the interaction of cellular CD153  [ **TNFSF8** ]  with immobilized CD30  [ **TNFRSF8** ] .  Ref: 11754357 Eur J Immunol, 2002
- Recombinant CD30L  [ **TNFSF8** ]  bearing cells up regulated the expression of surface CD30  [ **TNFRSF8** ]  and increased the production of IL 4 and soluble s CD30  [ **TNFRSF8** ]  by co cultured T cells.  Ref: 11728464 FEBS Lett, 2001
- PURPOSE Cutaneous anaplastic large cell lymphomas ALCLs are characterized by the expression of CD30  [ **TNFRSF8** ] , spontaneous regression of skin lesions, and increased concentration of CD30  [ **TNFRSF8** ]  ligand  [ **TNFSF8** ]  CD30L  [ **TNFSF8** ] .  Ref: 12855655 Clin Cancer Res, 2003

**Non-interactive relationship** (e.g. studied together, co-existance, homology, etc.)

- In addition the absence of CD30  [ **TNFRSF8** ]  L  [ **TNFSF8** ]  abolished clonal contraction after primary expansion and interfered with secondary expansion upon boosting.  Ref: 12538158 Ann N Y Acad Sci, 2002
- Association study between CD30  [ **TNFRSF8** ]  and CD30  [ **TNFRSF8** ]  ligand  [ **TNFSF8** ]  genes and type 1 diabetes in the Japanese population.  Ref: 11960307 Genes Immun, 2002
- Because transforming growth factor beta TGF beta and CD30  [ **TNFRSF8** ]  ligand  [ **TNFSF8** ]  inhibit the growth of normal lymphocytes and can be detected in regressing lesions of LyP, we tested the effect of these cytokines on cell lines clonally derived from LyP in the progression to systemic lymphoma.  Ref: 11594583 Ann N Y Acad Sci, 2001
- CD30  [ **TNFRSF8** ] , a member of the tumor necrosis factor  [ **TNFSF8** ]  receptor TNFR family, is a characteristic cell surface receptor for activated T cells and the malignant cells of Hodgkin s disease HD, anaplastic large cell lymphoma ALCL and a few other non Hodgkin s lymphomas.  Ref: 12389614 Leuk Lymphoma, 2002
- Co expression of interleukin 6 IL 6 and interleukin 6 receptor IL 6R in thyroid nodules is associated with co expression of CD30  [ **TNFRSF8** ]  ligand  [ **TNFSF8** ]  CD30  [ **TNFRSF8** ]  receptor.  Ref: 12553555 J Endocrinol Invest, 2002
- To investigate the roles of the co stimulatory molecules CD30  [ **TNFRSF8** ]  CD30L  [ **TNFSF8** ] , CD27 CD27L, OX40 OX40L, and 4 1BB 4 1BBL, which belong to the tumor necrosis factor  [ **TNFSF8** ]  TNF receptor ligand superfamily, in the development of acute viral myocarditis, the expression of these molecules was first analysed in the hearts of mice with acute myocarditis induced by Coxsackievirus B3 CVB3 in vivo.  Ref: 11745696 J Pathol, 2001
- Functional expression of receptor activator of nuclear factor kappaB in Hodgkin disease cell lines.The malignant Hodgkin and Reed Sternberg cells of Hodgkin disease express several members of the tumor necrosis factor  [ **TNFSF8** ]  receptor family, including CD30  [ **TNFRSF8** ]  and CD40, and secrete several cytokines and chemokines.  Ref: 11675352 Blood, 2001
- Based on our recent data of CD30  [ **TNFRSF8** ]  ligand  [ **TNFSF8** ]  CD30L  [ **TNFSF8** ]  CD30  [ **TNFRSF8** ]  receptor CD30  [ **TNFRSF8** ]  in these nodules and on the knowledge that this signal stimulates IL 6 production in non thyroid neoplasms, we wanted to evaluate the immunocytochemical expression of these 2 ligand receptor systems in a large archival series of paraffin embedded specimens.  Ref: 12553555 J Endocrinol Invest, 2002
- Because these foreign proteins are highly immunogenic in humans, we have developed a new CD30  [ **TNFRSF8** ]  ligand  [ **TNFSF8** ]  based fusion toxin Ang CD30L  [ **TNFSF8** ]  using the human RNase angiogenin.  Ref: 11751393 Cancer Res, 2001
- These findings were confirmed with AML blasts expressing surface CD30L  [ **TNFSF8** ] , where blocking anti CD30  [ **TNFRSF8** ]  antibodies completely abolished the release of sCD30 and reduced the production of IL 4.  Ref: 11728464 FEBS Lett, 2001
- Pleiotropic signal transduction mediated by human CD30  [ **TNFRSF8** ]  a member of the tumor necrosis factor  [ **TNFSF8** ]  receptor TNFR family.  Ref: 12389614 Leuk Lymphoma, 2002
- We conclude that only in a subset of both benign and malignant thyroid nodules the IL 6 IL 6R signal could be induced by the CD30L  [ **TNFSF8** ]  CD30  [ **TNFRSF8** ] .  Ref: 12553555 J Endocrinol Invest, 2002
- Cowpox virus encodes a fifth member of the tumor necrosis factor  [ **TNFSF8** ]  receptor family a soluble, secreted CD30  [ **TNFRSF8** ]  homologue.  Ref: 12034885 Proc Natl Acad Sci U S A, 2002
- Progression of lymphomatoid papulosis to systemic lymphoma is associated with escape from growth inhibition by transforming growth factor beta and CD30  [ **TNFRSF8** ]  ligand  [ **TNFSF8** ] .  Ref: 11594583 Ann N Y Acad Sci, 2001
- The tumor necrosis factor  [ **TNFSF8** ]  receptor family molecule CD30  [ **TNFRSF8** ]  is expressed by activated and memory T cells, depending on IL 4 stimulation preferentially in association with Th0 and Th2 type responses.  Ref: 11756152 Blood, 2002
- The purpose of this study was to determine whether CD30  [ **TNFRSF8** ]  or CD30L  [ **TNFSF8** ]  genes serve as a novel susceptibility gene for type 1 diabetes in humans.  Ref: 11960307 Genes Immun, 2002
- Co expression of IL 6 and IL 6R in the epithelial follicular cells was observed solely in CD30L  [ **TNFSF8** ]  CD30  [ **TNFRSF8** ]  positive nodules 5 15 33% oncocytic adenomas.  Ref: 12553555 J Endocrinol Invest, 2002
- All 3 non metastasized MTC were IL 6 IL 6R negative, and 1 3 was CD30L  [ **TNFSF8** ]  positive CD30  [ **TNFRSF8** ]  negative.  Ref: 12553555 J Endocrinol Invest, 2002
- Plasma levels of cytomegalovirus CMV specific immunoglobulin G IgG, soluble s CD30  [ **TNFRSF8** ] , sCD26 dipeptidyl peptidase IV DPP IV enzyme activity, and tumor necrosis factor  [ **TNFSF8** ]  receptor I TNFR I were assessed in human immunodeficiency virus HIV infected patients who experienced CMV retinitis CMVR as an immune restoration disease IRD during their first 6 months of highly active antiretroviral therapy HAART and in CMV seropositive, HIV infected patients with similar baseline CD4 T cell counts who had uneventful immune reconstitution.  Ref: 12085331 J Infect Dis, 2002
- In the normal thyroid tissue, colloid nodules, and another subset of benign and malignant thyroid nodules, IL 6 expression is under control of signals other than CD30L  [ **TNFSF8** ]  CD30  [ **TNFRSF8** ] .  Ref: 12553555 J Endocrinol Invest, 2002
- The notion that IL 4 was able to enhance in vitro proliferation of CD30L  [ **TNFSF8** ]  IL 4R purified leukaemic blasts suggests that the selective interaction of IL 4 producing CD30  [ **TNFRSF8** ]  T cells with CD30L  [ **TNFSF8** ]  leukaemic progenitors may have a role in the progression of this particular AML subset.  Ref: 11918534 Br J Haematol, 2002
- Unlike dendritic cells, the CD4 CD3 cells have little CD80 or CD86 expression but do express high levels of the TNF ligands, OX40 ligand and CD30  [ **TNFRSF8** ]  ligand  [ **TNFSF8** ] .  Ref: 12753741 Immunity, 2003
- 14 15 FTC and 5 6 ATC were CD30L  [ **TNFSF8** ]  CD30  [ **TNFRSF8** ]  negative.  Ref: 12553555 J Endocrinol Invest, 2002
- Characterization of the murine CD30  [ **TNFRSF8** ]  ligand  [ **TNFSF8** ]  CD153  [ **TNFSF8** ]  gene gene structure and expression.  Ref: 12392508 Tissue Antigens, 2002
- CD30  [ **TNFRSF8** ]  ligand  [ **TNFSF8** ]  CD30L  [ **TNFSF8** ] , but not its cognate receptor CD30  [ **TNFRSF8** ] , is frequently expressed on acute myeloid leukaemia AML blasts.  Ref: 11918534 Br J Haematol, 2002
- The affinity and kinetics of the interaction between soluble CD30  [ **TNFRSF8** ]  and CD153  [ **TNFSF8** ]  were determined using the BIAcore biosensor.  Ref: 11754357 Eur J Immunol, 2002
- We hypothesize that CD30  [ **TNFRSF8** ]  CD30L  [ **TNFSF8** ]  interactions explain the unusual clinical behavior of cutaneous ALCLs.  Ref: 12855655 Clin Cancer Res, 2003
- The production of IL 4 in the absence of interferon gamma IFN gamma was also detected in CD3 CD30  [ **TNFRSF8** ]  T cells from CD30L  [ **TNFSF8** ]  AML.  Ref: 11918534 Br J Haematol, 2002
- Creating CD30  [ **TNFRSF8** ]  L  [ **TNFSF8** ]  deficient mice and studying CD8 CTL activation and memory responses, it was observed that the absence of CD30  [ **TNFRSF8** ]  L  [ **TNFSF8** ]  resulted in diminished primary clonal expansion of CD8 cells.  Ref: 12538158 Ann N Y Acad Sci, 2002
- The immunoregulatory roles of interleukin 2 IL 2, IL 4, IL 10, gamma interferon IFN gamma, tumor necrosis factor  [ **TNFSF8** ]  alpha TNF alpha, the soluble form of the IL 2 receptor sIL 2R, and the soluble form of CD30  [ **TNFRSF8** ]  sCD30 were evaluated in patients with hepatitis B virus HBV infection.  Ref: 12414777 Clin Diagn Lab Immunol, 2002
- IL 6 staining associated to absent expression of CD30L  [ **TNFSF8** ]  and CD30  [ **TNFRSF8** ]  was observed in 7 follicular adenomas all belonging to variants different from toxic and hyalinizing trabecular, 2 oncocytic adenomas, 5 of the 30 colloid nodules and 2 normal thyroids.  Ref: 12553555 J Endocrinol Invest, 2002
- CD153  [ **TNFSF8** ]  CD30  [ **TNFRSF8** ]  ligand  [ **TNFSF8** ]  has been described as a 40 kDa type II transmembrane glycoprotein belonging to the TNF superfamily and is expressed primarily by activated T cells, B cells and monocytes.  Ref: 12392508 Tissue Antigens, 2002
- We show that vCD30 is a 12 kD secreted protein that not only binds CD30L  [ **TNFSF8** ]  with high affinity and prevents its interaction with CD30  [ **TNFRSF8** ] , but it also induces reverse signaling in cells expressing CD30L  [ **TNFSF8** ] . vCD30 blocked the generation of interferon gamma producing cells in vitro and was a potent inhibitor of T helper cell Th 1 but not Th2 mediated inflammation in vivo.  Ref: 12235215 J Exp Med, 2002
- In conclusion, we could not find significant association between CD30  [ **TNFRSF8** ]  or CD30L  [ **TNFSF8** ]  genes and type 1 diabetes, but CA 9 allele in the promotor or T allele of 73T C in intron 3 in CD30L  [ **TNFSF8** ]  gene might play a minor role in the pathogenesis of type 1 diabetes, only in the Japanese female population.  Ref: 11960307 Genes Immun, 2002
- In a cell kinetic analysis we found that ALCL cell growth is effectively inhibited by CD30L  [ **TNFSF8** ]  but only in those cell lines expressing CD30  [ **TNFRSF8** ]  molecules in sufficient amounts on the cell surface.  Ref: 12855655 Clin Cancer Res, 2003
- The results suggest that progression of LyP to lymphoma is associated with escape of lymphoma cells from growth regulation by TGF beta and CD30  [ **TNFRSF8** ]  ligand  [ **TNFSF8** ] .  Ref: 11594583 Ann N Y Acad Sci, 2001
- We screened CD30  [ **TNFRSF8** ]  and CD30L  [ **TNFSF8** ]  genes for polymorphisms in Japanese patients with type 1 diabetes and control subjects.  Ref: 11960307 Genes Immun, 2002
- A vCD30Fc fusion protein binds CD153  [ **TNFSF8** ]  CD30  [ **TNFRSF8** ]  ligand  [ **TNFSF8** ]  specifically, and it completely inhibits CD153  [ **TNFSF8** ]  CD30  [ **TNFRSF8** ]  interactions.  Ref: 12034885 Proc Natl Acad Sci U S A, 2002
- Soluble CD30  [ **TNFRSF8** ]  may bind to its ligand, CD153  [ **TNFSF8** ] , with high affinity and block its interaction with membrane bound CD30  [ **TNFRSF8** ] .  Ref: 11754357 Eur J Immunol, 2002
- Association studies revealed no association between the CD30  [ **TNFRSF8** ]  and CD30L  [ **TNFSF8** ]  genes and type 1 diabetes in the whole population.  Ref: 11960307 Genes Immun, 2002
- The authors have reported previously that the B lymphocytes of HD express CD30  [ **TNFRSF8** ]  ligand  [ **TNFSF8** ]  CD30L  [ **TNFSF8** ] , CD153  [ **TNFSF8** ] .  Ref: 11815953 Cancer, 2002
- CD30  [ **TNFRSF8** ]  CD30  [ **TNFRSF8** ]  ligand  [ **TNFSF8** ]  CD30L  [ **TNFSF8** ]  signal transduction appears to protect against autoimmune diabetes by preventing expansion of autoreactive T cells and suppressing Th1 cytokine response.  Ref: 11960307 Genes Immun, 2002
- Of the 6 tumors arising from the parafollicular C cells medullary thyroid cancer, MTC, all 3 that had metastasized were CD30L  [ **TNFSF8** ]  CD30  [ **TNFRSF8** ]  IL 6 positive and IL 6R negative.  Ref: 12553555 J Endocrinol Invest, 2002
- A CD30  [ **TNFRSF8** ]  ligand  [ **TNFSF8** ]  agonist antibody caused proliferation of tumor cells from one patient and had no effect on tumor cells of another.  Ref: 11594583 Ann N Y Acad Sci, 2001
- In another oncocytic adenoma, another 4 conventional PTC and another 7 non conventional PTC CD30L  [ **TNFSF8** ]  CD30  [ **TNFRSF8** ]  expression was associated to expression of IL 6 only.  Ref: 12553555 J Endocrinol Invest, 2002
